# Experimental infectious challenge in pigs leads to elevated fecal calprotectin levels following colitis, but not enteritis

**DOI:** 10.1186/s40813-021-00228-9

**Published:** 2021-08-24

**Authors:** Jéssica A. Barbosa, Lucas A. Rodrigues, Daniel A. Columbus, Juan C. P. Aguirre, John C. S. Harding, Vinícius S. Cantarelli, Matheus de O. Costa

**Affiliations:** 1 Animal Science Department, Federal University of Lavras, Lavras, Minas Gerais 37200-000 Brazil; 2 Prairie Swine Centre, Inc., 2105 - 8th Street East, PO Box 21057, Saskatoon, SK S7H 5N9 Canada; 3 Department of Animal and Poultry Science, University of Saskatchewan, 51 Campus Dr, Saskatoon, SK S7N 5A8 Canada; 4 Department of Large Animal Clinical Sciences, Western College of Veterinary Medicine, University of Saskatchewan, 52 Campus Dr, Saskatoon, SK S7N 5B4 Canada; 5 Department of Population Health Sciences, Faculty of Veterinary Medicine, Utrecht University, Yalelaan 7, Utrecht, 3584 CL The Netherlands

**Keywords:** Biological markers, Intestinal inflammation, Enteric disease, Swine

## Abstract

**Background:**

Fecal calprotectin is largely applied as a non-invasive intestinal inflammation biomarker in human medicine. Previous studies in pigs investigated the levels of fecal calprotectin in healthy animals only. Thus, there is a knowledge gap regarding its application during infectious diarrhea. This study investigated the usefulness of fecal calprotectin as a biomarker of intestinal inflammation in *Brachyspira hyodysenteriae* and *Salmonella* Typhimurium infected pigs.

**Results:**

Fecal samples from pigs with colitis (*n* = 18) were collected from animals experimentally inoculated with *B. hyodysenteriae* (*n* = 8) or from sham-inoculated controls (*n* = 3). Fecal samples from pigs with enteritis (*n* = 14) were collected from animals inoculated with *Salmonella enterica* serovar Typhimurium (*n* = 8) or from sham-inoculated controls (*n* = 4). For both groups, fecal samples were scored as: 0 = normal; 1 = soft, wet cement; 2 = watery feces; 3 = mucoid diarrhea; and 4 = bloody diarrhea. Fecal calprotectin levels were assayed using a sandwich ELISA, a turbidimetric immunoassay and a point-of-care dipstick test. Fecal calprotectin levels were greater in colitis samples scoring 4 versus ≤ 4 using ELISA, and in feces scoring 3 and 4 versus ≤ 1 using immunoturbidimetry (*P* < 0.05). No differences were found in calprotectin concentration among fecal scores for enteritis samples, regardless of the assay used. All samples were found below detection limits using the dipstick method.

**Conclusions:**

Fecal calprotectin levels are increased following the development of colitis, but do not significantly change due to enteritis. While practical, the use of commercially available human kits present sensitivity limitations. Further studies are needed to validate the field application of calprotectin as a marker of intestinal inflammation.

**Supplementary Information:**

The online version contains supplementary material available at 10.1186/s40813-021-00228-9.

## Background

The use of antimicrobials as growth promoters (APG) in pork production has been globally discouraged due to the emergence of multi-drug resistant bacterial strains which can impose risks to human and animal health [1, 2]. In most countries with significant pork production, the current policies on the use of antimicrobial agents have resulted in a need for improved on-farm biosecurity, nutritional, husbandry, and welfare practices, as well as the development of tools to guide the use of antimicrobials [3]. A non-invasive biomarker for intestinal inflammation would result in more judicious therapeutic and nutritional interventions during episodes of enteric diseases in commercial operations.

Swine dysentery (SD) and porcine salmonellosis are intestinal disorders of global relevance in grower-finisher pigs. Both diseases are associated with significant economic losses due to increased production costs and poor animal performance [4, 5]. Mucohemorrhagic diarrhea and colitis (inflammation of the large intestine) are the main clinical signs of SD caused by *Brachyspira hyodysenteriae*. *B. hampsonii* and *B. suanatina* [6]. Currently, the use of antimicrobials is the only strategy to prevent and treat this disease [7]. *Salmonella enterica* serovar Typhimurium causes enteritis (inflammation of the small intestine) and watery diarrhea in pigs [5, 8]. Even though studies have evaluated vaccination to control salmonellosis in pigs, protection is variable due to poor cross-protection across serovars [9, 10], and antimicrobials are still used metaphylactically.

Calprotectin is a 24 kDa calcium binding protein of the S100 family. It accounts for approximately 60% of the cytosolic protein in neutrophils and is also found in monocytes [11, 12]. It is released upon neutrophil activation and displays antimicrobial, antiproliferative and apoptotic properties [12, 13]. Interestingly, calprotectin is resistant to intestinal bacteria proteases [14]. In human medicine, calprotectin has been used to assess the extent of intestinal inflammation [15]. Its concentration in feces is correlated with inflammatory bowel disease (IBD) [16, 17], and necrotic enterocolitis in infants [18]. Fecal calprotectin is used to identify and aids in distinguishing IBD from irritable bowel syndrome (IBS) [19, 20], and is specifically useful to predict disease activity and relapse during treatment [21, 22]. Increased fecal calprotectin levels were associated with endoscopic and histological lesions during episodes of IBD [23, 24] and can be used to distinguish between inflammatory and non-inflammatory colitis in humans [25]. Physicians often apply this concept to distinguish IBD relapses from true infectious colitis and diarrhea [21, 25]. Thus, there is a plethora of commercially available kits aimed at detecting human calprotectin in feces, ranging from laboratory-intensive ELISAs to point-of-care dipsticks.

Studies focused on swine have investigated calprotectin levels in the feces of healthy animals only, suggesting it may be involved in intestinal homeostasis [26, 27]. However, there are no reports on the use of calprotectin as a biomarker of intestinal inflammation in disease-challenged pigs. We hypothesized that, similar to what is observed in humans, pigs with intestinal inflammation have increased levels of fecal calprotectin. The swine calprotectin S100-A8 subunit amino-acid sequence is 72% similar to the human protein, and the S100-A9 subunit is 66% similar. Thus, we also hypothesized that commercial kits aimed at human calprotectin should also detect the swine protein. Therefore, the objective of this study was to evaluate the usefulness of fecal calprotectin as a biomarker of colitis or enteritis in swine using commercially available human kits.

## Results

### Colitis samples assessment

**Fig. 1:**
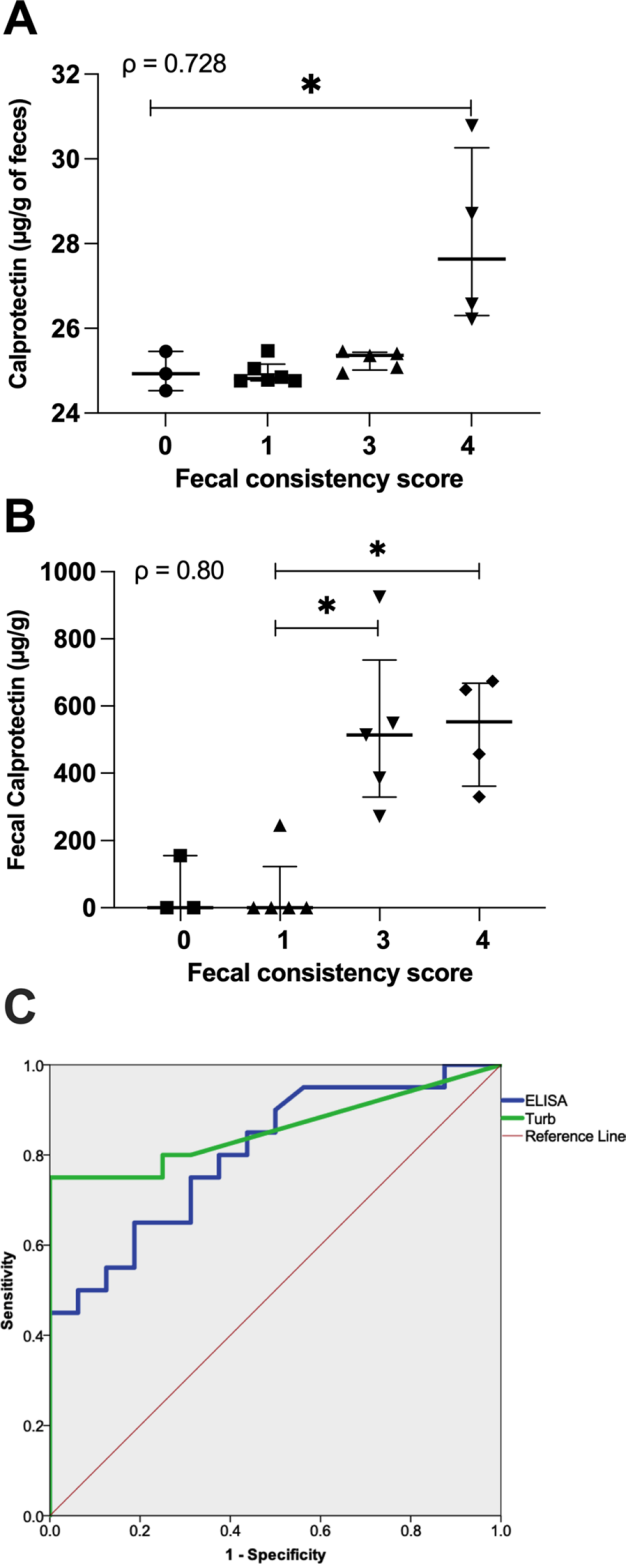
Calprotectin concentration in colitis fecal samples (COL, µg/g) from pigs challenged with B. *hyodysenteriae*. **A** ELISA assay; **B** Immunoturbidimetry assay; **C** ROC curve analysis plot (Turb—immunoturbidimetry assay). Stars denote a significant difference (*P* < 0.05) between fecal scores. Bars denote median, with interquartile range shown error bars. (ρ = spearman’s correlation coefficient)

Using ELISA, fecal samples that scored 4 (bloody diarrhea) had higher calprotectin levels than those that scored 0, 1 or 3 (*P* = 0.037, Fig. 1A). Using immunoturbidimetry, fecal samples that scored 3 and 4 had higher calprotectin levels than those that scored 1 (score 3 *P* = 0.039, score 4, *P* = 0.044 respectively, Fig. 1B). Fecal calprotectin level was positively correlated with fecal consistency scores using ELISA (ρ = 0.728; *P* = 0.001, Fig. 1A) and immunoturbidimetry (ρ = 0.80; *P* = 0.001, Fig. 1B). ELISA was positively correlated with the immunoturbidimetry assay (ρ = 0.55; *P* = 0.017). ROC curve analysis (Fig. 1C) revealed that both ELISA (*P* = 0.002) and immunoturbidimetry (*P* = 0.000) could reliably diagnose a diseased state. Immunochromatographic dipstick tested negative for all samples.

### Enteritis samples assessment

**Fig. 2:**
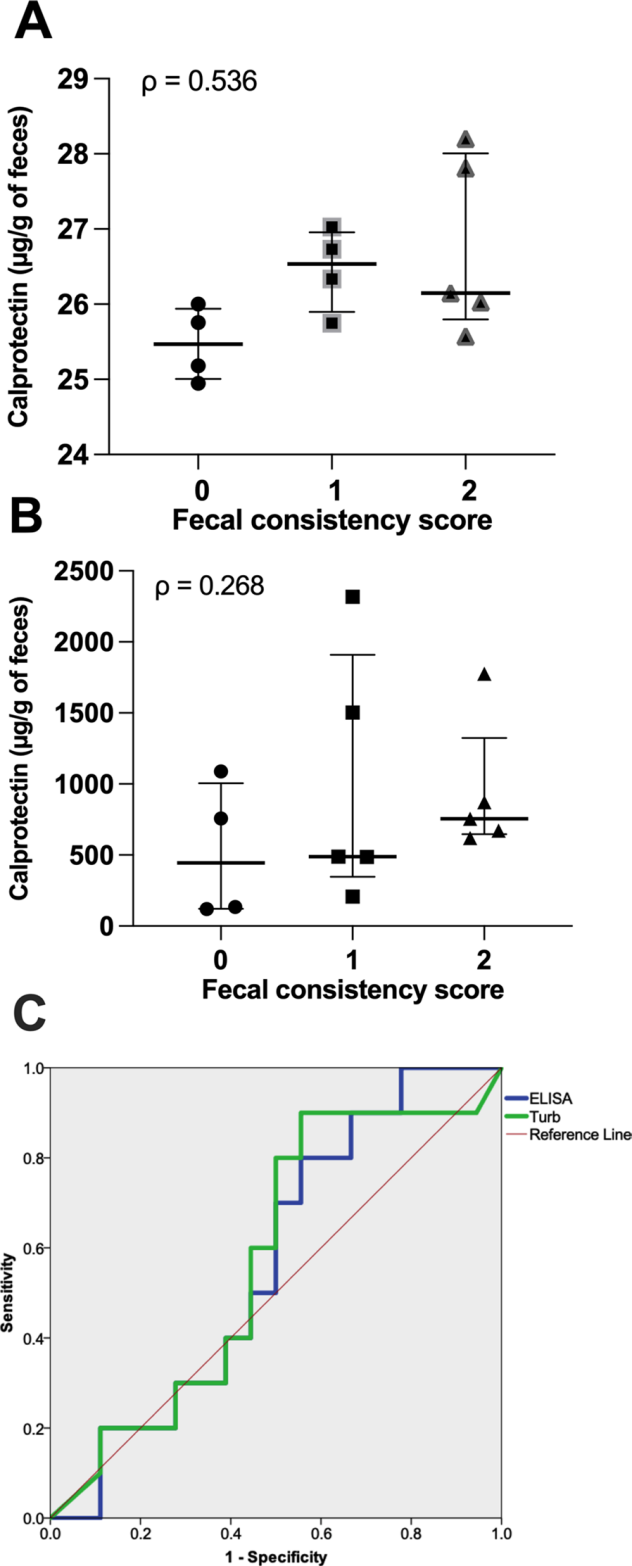
Calprotectin concentration in enteritis fecal samples (ENT, µg/g) from pigs challenged with *S*. Typhimurium. **A** ELISA assay; **B** Immunoturbidimetry assay. **C** ROC curve analysis plot (Turb—immunoturbidimetry assay). Stars denote a significant difference (*P* < 0.05) between fecal scores. Bars denote median, with interquartile range shown error bars. (ρ = spearman’s correlation coefficient)

No differences were found in calprotectin concentration among fecal score groups when measured using ELISA (*P* = 0.098; Fig. 2A) or immunoturbidimetry (*P* = 0.579; Fig. 2B). However, fecal scores 1 and 2 did have numerically higher fecal calprotectin concentrations than score 0 using either method. Fecal calprotectin concentration was not correlated with fecal consistency scores when analyzed by either ELISA (ρ = 0.536; *P* = 0.59; Fig. 2A) or immunoturbidimetry (ρ = 0.268; *P* = 0.376; Fig. 2B). The same correlation pattern was observed between ELISA and Immunoturbidimetry assays (ρ = 0.464; *P* = 0.095). ROC curve analysis (Fig. 2C) revealed no statistical significance regarding the ability of either ELISA (*P* = 0.56) or immunoturbidimetry (*P* = 0.51) assays in diagnosing a diseased state. Additionally, all samples tested negative when the immunochromatographic dipstick test was used.

## Discussion

Grower-finisher infectious diarrhea in commercial swine operations leads to decreased performance and increased production costs associated with treatment and mortality, directly impacting profits [4, 5]. To help direct immediate therapeutic and nutritional interventions following observation of diarrhea, a non-invasive intestinal inflammation biomarker test to differentiate inflammatory from non-inflammatory causes of diarrhea would be beneficial for practitioners. In this study, we observed that fecal calprotectin levels, measured by ELISA or immunoturbidimetry, increases following the development of colitis and mucoid or bloody diarrhea in pigs challenged with *B. hyodysenteriae.* However, we did not find any changes in fecal calprotectin levels due to enteritis caused by *S.* Typhimurium. Both methodologies were ineffective in discerning between mild, watery diarrhea and normal feces during colitis or enteritis.

Calprotectin is a calcium binding protein secreted by neutrophilic granulocytes and has a role controlling bacterial growth during inflammation [12, 28]. Recruitment of neutrophils to the intestinal mucosa leads to neutrophil cell shedding and active secretion of calprotectin to the intestinal lumen [12]. Currently, recognized triggers of calprotectin secretion are lipopolysaccharide and monosodium ureate [1, 2]. This is in line with findings showing that, in humans, bacterial agents lead to higher fecal calprotectin levels than viral [41, 56, 57]. Once secreted, calprotectin sequesters essential micronutrients such as iron, zinc, and manganese, inhibiting bacterial growth [29, 30]. Fecal calprotectin concentration has been shown to be correlated with the number of neutrophils released in the intestinal lumen during inflammation, which in humans can be associated with the severity of inflammation [19]. Previous studies investigating calprotectin levels in the feces of healthy pigs suggested it may play a role in intestinal homeostasis [26]. Lallès et al. [27] observed that the average fecal calprotectin concentration from sow samples (13 ± 38 mg/kg of feces) was close to the range described in healthy human adults (range 2–47 mg/kg), but the concentrations found from piglet samples at birth were lower (24 ± 60 mg/kg) than human newborns (145 ± 78.5 mg/kg). The same authors also found very low fecal calprotectin levels in healthy pigs under high sanitary conditions. Elevated fecal calprotectin is a common finding in humans with IBD [16, 22]. In humans, patients with IBD and IBS have similar clinical signs. Calprotectin is already extensively used in human medicine as a biomarker of IBD, as it can help distinguish IBS from IBD, and detect recurrent IBD during treatment [20, 25]. Fecal calprotectin levels reported from dog samples can be used to discern between animals with different causes of chronic inflammatory enteropathies such as steroid‐responsive/refractory enteropathy and immunosuppressant‐responsive/‐refractory enteropathy, and animals with food‐responsive enteropathy or antibiotic‐responsive enteropathy before treatment [31, 32].

Here elevated fecal calprotectin levels in pigs were associated with mucoid or haemorrhagic colitis, but not enteritis. While further studies using larger populations are needed to validate these results, our data suggests that fecal calprotectin could be a potential tool used to diagnose severe inflammatory colitis, particularly by untrained observers who may, for example, miss blood staining in feces when pigs are housed in large groups. It may also help distinguish bacterial colitis from other causes of diarrhea in pigs, thus, contributing to a more judicious use of antimicrobials for pork production. We found that mucoid or mucohemorragic feces from pigs with colitis contained the highest calprotectin concentration. Multiple previous reports have characterized the accumulation of neutrophils, a source of antimicrobial peptides such as calprotectin, on the surface of the colonic mucosa during *B. hyodysenteriae* and *B. hampsonii* infection in pigs [33–36]. Here we found evidence that severe SD clinical signs are associated with increased fecal calprotectin levels, providing further evidence of the importance of neutrophils in the pathogenesis of swine dysentery.

In swine, *S.* Typhimurium invades epithelial cells of the small intestine. It can invade colonocytes as well, leading to inflammatory diarrhea with a marked increase in mucosal neutrophil infiltration [5, 8, 37]. Despite this, we did not observe a significant increase in fecal calprotectin levels following inoculation with *S.* Typhimurium, regardless of the assay used. Our findings differ from previous studies that found increased fecal calprotectin concentration during *S.* Typhymurium infection in rats [38, 39], and *Salmonella* spp. infection in humans [40]. Human patients with severe or moderate bacterial gastroenteritis and fecal mucus have increased fecal calprotectin, but those with mild diarrhea do not [41]. Mucoid feces is not a feature of swine salmonellosis, but it is associated with *Brachyspira* spp. [5]. Moreover, it has been shown that *S.* Typhimurium overcomes the antimicrobial effect of calprotectin by expressing a high affinity zinc transporter (ZnuABC) [39, 42]. We recognize that the lack of histopathology data from either sample cohort is a limitation here and suggest the collection of such samples in future studies.

The literature is contradictory regarding the association between high fecal calprotectin levels and lesion site. There are reports that either ileal or colonic lesions can both be monitored using fecal calprotectin as an indicator of endoscopically active Crohn’s disease (CD) [16, 43–45]. In contrast, other studies have found that the discriminatory power of fecal calprotectin is greater in ileocolonic and colonic CD, than in jejunal or ileal CD [46–48]. Zittan et al. [47] postulated that the slow intestinal transit in the colon could increase calprotectin degradation through intestinal proteases, thereby reducing its concentration in feces. We believe that the lack of difference in calprotectin levels in enteritis samples was due to the proximal location of the lesions, which were most likely associated with the small intestine [49]. Differently from humans, pigs have a functional cecum that may contribute to this disappearance effect by luminal proteases. Furthermore, age may as well impact luminal calprotectin clearance. The gastrointestinal tract length of pigs used in this study were a portion of the size of a finisher pig, together with the functional changes that take place following weaning these could be factors that influence the disappearance of calprotectin released in the small intestine.

Interestingly, higher concentrations of fecal calprotectin were found when measured using the immunoturbidimetry assay compared to ELISA in both sample cohorts. For human samples, ELISA based on monoclonal antibodies is the gold standard used to quantify fecal calprotectin levels. It is specific to calprotectin heterodimeric and polymeric complexes. However, ELISA is laborious and time-consuming [13, 50] when compared to the a particle enhanced turbidimetric immunoassays (PETIA), based on polystyrene nanoparticles coated with calprotectin-specific antibodies binding to their specific target within the extracted samples. Subsequent quantification of the agglutinated calprotectin-nanoparticle complex detected by light absorbance (turbidity) can be adapted to several commercially available clinical chemistry analyzers and has been proposed as a rapid response test [51]. Labaere et al. [52] compared different calprotectin detection methods (three rapid quantitative immunochromatografic tests, two enzyme-linked immunosorbent assays, and one automated fluoroimmunoassay), and reported significant variations in the calprotectin levels detected. Juricic et al. [53] reported fecal calprotectin concentrations using a commercial ELISA kit to be significantly lower than a turbidimetric immunoassay. Oyaert et al. [54] observed satisfactory diagnostic performance between six different fecal calprotectin immunoassays (two ELISA, two chemiluminescent immunoassays (CLIA), one fluoroenzyme immunoassay (FEIA), and one PETIA), even though there were discrepancies in calprotectin values detected between these kits. These reports are consistent with our findings that different assays resulted in different values for fecal calprotectin. It is worth mentioning that the kits evaluated in this study used monoclonal antibodies specific for human calprotectin, therefore, the low calprotectin levels found by ELISA may be due to the lack of cross reactivity with the swine protein, as previously reported [26]. While we understand the limitation of this approach, commercial kits for fecal calprotectin detection are only available for humans. In addition, there are multiple point-of-care kits commercially available that could be translated into farm-friendly tools. Nevertheless, we still found evidence that human tests can be used in veterinary medicine, taking advantage of this previously developed infrastructure. However, test sensitivity must be further evaluated and optimized for swine, if deemed necessary by future investigations. We recognize that there are multiple other causes of enteritis and colitis in pigs, we believe that *B. hyodysenteriae* and *S.* Typhimurium are also representative of these syndromes. We also appreciate that a limited number of samples were utilized in both COL and ENT groups. This likely limited some of our findings related to the less severe fecal scores.

## Conclusions

This initial data suggests that fecal calprotectin only peaks to detectable levels following colitis, but not enteritis. The approach used was unable to discern between mild-diarrhea and healthy feces, or when pigs only developed enteritis*.* Further investigations are suggested as this approach has the potential to support the judicious use of antimicrobials for pork production through the differentiation of infectious from non-infectious causes of colitis.

## Methods

### Animal trials and fecal samples

Two independent trials (one for each pathogen) were performed where pigs were obtained from the same PRRSV negative, high-health herd with no gastrointestinal clinical signs and historically free from swine dysentery and salmonellosis. Animals were housed and allowed to acclimate in a BSL-2 animal care facility for 7 days prior to inoculation. Colitis samples (COL, *n* = 18) were obtained from 9-to-10-week-old barrow pigs (housed in pens with 6 pigs/pen) experimentally inoculated (*n* = 8) thrice over 72 h with *Brachyspira hyodysenteriae* G44 (obtained from a clinical case), the etiologic agent of swine dysentery, or from sham-inoculated controls (*n* = 3). A commercial starter diet, unmedicated, fed ad libitum was used. Pigs were intragastrically inoculated with 50 mL liquid media averaging 1.69 × 10^9^ genome equivalents/mL as previously described [35]. A summary of the samples used from this trial is shown on Table 1. The development of swine dysentery was confirmed by associating clinical signs, positive fecal *B. hyodysenteriae* culture and gross necropsy lesions (data not shown). Enteritis samples (ENT, *n* = 14) were collected from pigs experimentally inoculated with *Salmonella enterica* serovar Typhimurium var Copenhagen (*n* = 9, isolated from a clinical case), or from non-infected controls (*n* = 4). After a 7-days acclimation period, pigs were orally inoculated twice within 4 h with 1 mL containing 3.3 × 10^9^ CFU/mL/pig of *S.* Typhimurium, as previously described [55], or 1 mL of sterile saline solution (non-infected controls). Pigs were fed a diet that met the minimum requirements for this age, and were group housed in pens with 8 animals [55]. Sample summary is also shown in Table 1. All animals tested negative by culture for their inoculation agent upon arrival at the BSL-2 facility [35, 55]. Daily monitoring for pathogen of interest shedding was also performed as previously described [35, 55], and only positive samples were used in this study. Briefly, *Brachyspira* spp. culture was performed using BJ agar in anaerobic chambers at 42 °C for up to 10 days. *Salmonella* samples were cultured in brilliant green agar and verified by broth culture using enriched selenite-cysteine broth. As expected, fecal scores for this trial ranged from 0–2. The development of salmonellosis was confirmed by fecal culture, gross necropsy lesions (no signs of typhlitis or colitis were observed), clinical signs, intestinal levels of antioxidant enzymes and performance parameters (data not shown). Feces from both trials were collected following digital stimulation, and only one sample per pig per score was included. Scoring followed a previously developed fecal consistency rubric [35]: 0 = normal; 1 = soft, wet cement; 2 = watery feces; 3 = mucoid diarrhea; and 4 = bloody diarrhea. All fecal samples were obtained from individual pigs and stored at -80ºC until processing for analysis.

**Table 1:** Summary of fecal samples used in this study

**Fecal Score**	**Inoculation Group**	**Calprotectin group**	**Collection day (dpi)**
0	Control-SD	Colitis	0
0	Control-SD	Colitis	0
0	Control-SD	Colitis	5
1	SD	Colitis	9
1	SD	Colitis	6
1	SD	Colitis	8
1	SD	Colitis	8
1	SD	Colitis	5
2	SD	Colitis	5
3	SD	Colitis	5
3	SD	Colitis	5
3	SD	Colitis	15
3	SD	Colitis	8
3	SD	Colitis	9
4	SD	Colitis	7
4	SD	Colitis	10
4	SD	Colitis	5
4	SD	Colitis	8
0	Control-ST	Enteritis	2
0	Control-ST	Enteritis	3
0	Control-ST	Enteritis	5
0	Control-ST	Enteritis	5
1	ST	Enteritis	2
1	ST	Enteritis	1
1	ST	Enteritis	5
1	ST	Enteritis	5
1	ST	Enteritis	4
2	ST	Enteritis	2
2	ST	Enteritis	2
2	ST	Enteritis	3
2	ST	Enteritis	2
2	ST	Enteritis	2

*SD* Swine dysentery, samples from pigs inoculated with *B. hyodysenteriae*, *ST Salmonella* Typhimurium, samples from a pigs inoculated with *S.* Typhimurium, *Dpi* Days post-inoculation

### Fecal sample extraction

Fecal samples were processed according to the kit manufacturers’ instructions, with minor changes as described below (Bühlmann Calprotectin ELISA EK-CAL, Bühlmann Laboratories AG, Switzerland). For each sample, between 50 and 100 mg of feces were weighed into a sterile polypropylene tube (15 mL, VWR Scientific Products, Suwanee, GA, USA). Extraction buffer was added, adjusting the reaction volume to each sample weight to obtain a final 1:10 ratio. Extraction tubes were individually vortexed for 30 s (Fisher Vortex Genie 2, Fisher Scientific, Pittsburgh, PA, USA) at maximum speed and incubated for 30 min at room temperature on a shaker at 400 rpm (G-25 Incubator Shaker, New Brunswick Scientific Co., Inc., Edison, NJ, USA). Samples were vortexed again for 30 s, a 1.5 mL aliquot was transferred to a 2 mL sterile microfuge tube and centrifuged at 3000 g for 5 min. Finally, the supernatant was transferred to a 1.5 mL microfuge tube and stored at -20 °C until analysed.

### Enzyme-linked immunosorbent assay (ELISA)

ELISA was carried out following the manufacturer’s instructions (Bühlmann Calprotectin ELISA EK-CAL, Bühlmann Laboratories AG, Switzerland). Fecal extracts were thawed and homogenized prior to analysis. Initially, 100 µL of incubation buffer (blank, negative control), five calibrator samples (100 µL/well, ranging from 30 to 1800 µg/g; Additional file 1: Table 1), and low and high control samples (100 µL/well) were included on each microtiter plate precoated with anti-calprotectin monoclonal antibodies (mAb). Finally, 100 µL of fecal extract per sample was analyzed. All samples were analyzed in duplicates, including extraction controls. Following dispensing of samples and controls, reaction plates were incubated for 35 min using an orbital plate shaker at 450 rpm, at room temperature. After incubation, plates were washed three times for 30 s with 300 µL of wash buffer per well. Next, each sample was incubated and mixed for 35 min with 100 µL of enzyme label anti- mAb conjugated with horseradish peroxidase (HRP). The wash step was repeated 5 times as described above and immediately after; the color reaction was induced using 100 µL of tetramethylbenzidine (TBM). The plate was covered with a plate sealer (Bühlmann Laboratories AG, Switzerland) to prevent TBM degradation due to exposure to light, and incubated for 15 min on a plate shaker at 400 rpm at room temperature. The reaction was stopped by adding 100 µL of 0.25 M sulfuric acid to each well and absorbance assessed at 450 nm using a microplate reader (Biotek Epoch, Biotek Instruments, Winooski, Vermont, USA). Calprotectin level was expressed as micrograms per gram (μg/g) of feces and values are reported as the mean value for both duplicates.

### Immunoturbidimetry assay

Fecal extracts were thawed and analyzed using the fCal Turbo assay (BÜHLMANN, Laboratories AG, Switzerland). This assay was adapted to be performed on a plate reader. Reaction buffer (150 µL) and immunoparticles (30 µL) were pipetted into all wells of a test plate. Six calibrator samples (10 µL/well, ranging from 0 to 2207.6 µg/g; Additional file 1: Table 2) were included in each plate. Ten µL of fecal extract per sample was tested in duplicate. Absorbance was measured at 546–580 nm using a microplate reader (Biotek Epoch, Biotek Instruments, Winooski, Vermont, USA) using the Gen5 Data Analysis software interface (Biotek Instruments, Winooski, Vermont, EUA).

### Immunochromatographic assay

Samples were also analyzed using a point-of-care dipstick test for detection of calprotectin in feces (Actim calprotectin rapid test, Medix biochemica, Espoo, Finland) following the manufacture’s instructions. This is a semi-quantitative test with a detection range of 12.5 to 10,000 μg of calprotectin/g of human feces. Briefly, 1 g from each fecal sample was brought to room temperature and added to the dilution buffer container. The container was manually shaken, and the detection stick was inserted in the container once the sample was diluted. Results were read after 10 min contact between the test strip and the sample.

### Statistical analysis

One fecal sample from the COL group (the only score 2) was removed from the analyses but is still shown in the plots for visual comparison only. Analyses were performed using SPSS (IBM‐SPSS, Chicago, IL, USA). Differences in calprotectin levels among fecal score groups were analyzed using the Kruskal–Wallis test. When there was a significant overall group difference, the Dunn’s *post-hoc* test was used to assess pairwise differences. The association between calprotectin concentration and fecal consistency score, as well as between ELISA and Immunoturbidimetry assays, was assessed by determining the Spearman’s correlation coefficient (ρ). Alpha level for determination of significance was 0.05. A receiver operator characteristic (ROC) curve analysis was performed to assess the diagnostic efficiency of each diagnostic method. Fecal scores ≥ 2 were used as the clinical threshold for diarrhea (positive sample).

## Supplementary Information

**Additional file 1: Table 1.** Summary of calibrator and reaction control results for ELISA assays. **Table 2.** Summary of calibrator and reaction control results for Immunoturbidimetry assay.

**Additional file 2: Figure 1.** Representative H&E stained sections of formalin fixed colon (COL group) and ileum (ENT group) samples.

## Data Availability

Not applicable.

## Abbreviations

CD: Crohn’s disease
CLIA: Chemiluminescent immunoassay
BSL-2: Biosafety level 2
COL: Colitis samples
ELISA: Enzyme-linked immunosorbent assay
ENT: Enteritis samples
FEIA: Fluoroenzyme immunoassay
IBD: Inflammatory bowel disease
IBS: Irritable bowel syndrome
HRP: Horseradish peroxidase
mAb: Anti-calprotectin monoclonal antibodies
PETIA: Particle enhanced turbidimetric immunoassay
PRRSV: Porcine reproductive and respiratory syndrome virus
SD: Swine dysentery
TBM: Tetramethylbenzidine
ZnuABC: High affinity zinc transporter
