# Supplementary material for: Experimental infectious challenge in pigs leads to elevated fecal calprotectin levels following colitis, but not enteritis
Source: Porcine Health Manag. 2021 Aug 24;7:48. doi: 10.1186/s40813-021-00228-9 (PMC8383374; doi:10.1186/s40813-021-00228-9)
Supplement: Supplementary file 1 — Additional file 1: Table 1. Summary of calibrator and reaction control results for ELISA assays. Table 2. Summary of calibrator and reaction control results for Immunoturbidimetry assay. [file 40813_2021_228_MOESM1_ESM.docx]

**Table 1.** Summary of calibrator and reaction control results for ELISA assays

| **Reagents** | **Manufacturer predicted value (µg/g)** | **Measured value (n=2, µg/g)** |
| --- | --- | --- |
| Blank | N/A | 24.601 |
| Calibrator A | 30 | 30 |
| Calibrator B | 90 | 89.999 |
| Calibrator C | 300 | 300 |
| Calibrator D | 900 | 900.001 |
| Calibrator E | 1800 | 1800.031 |
| Low control | 66-171 | 127.436 |
| High control | 303-561 | 408.966 |

**Table 2.** Summary of calibrator and reaction control results for Immunoturbidimetry assay

| **Reagents** | **Manufacturer predicted value (µg/g)** | **Measured value (n=2, µg/g)** |
| --- | --- | --- |
| Calibrator 1 | 0 | 0 |
| Calibrator 2 | 55.3 | 0 |
| Calibrator 3 | 220.6 | 536.119 |
| Calibrator 4 | 552.3 | 1.127.235 |
| Calibrator 5 | 1104.1 | 1.399.032 |
| Calibrator 6 | 2207.6 | 1.892.208 |
| Low control | 68-104 | 0 |
| High control | 228-342 | 602.765 |
