# Supplementary figures and images for: Experimental infectious challenge in pigs leads to elevated fecal calprotectin levels following colitis, but not enteritis
Source: Porcine Health Manag. 2021 Aug 24;7:48. doi: 10.1186/s40813-021-00228-9 (PMC8383374; doi:10.1186/s40813-021-00228-9)

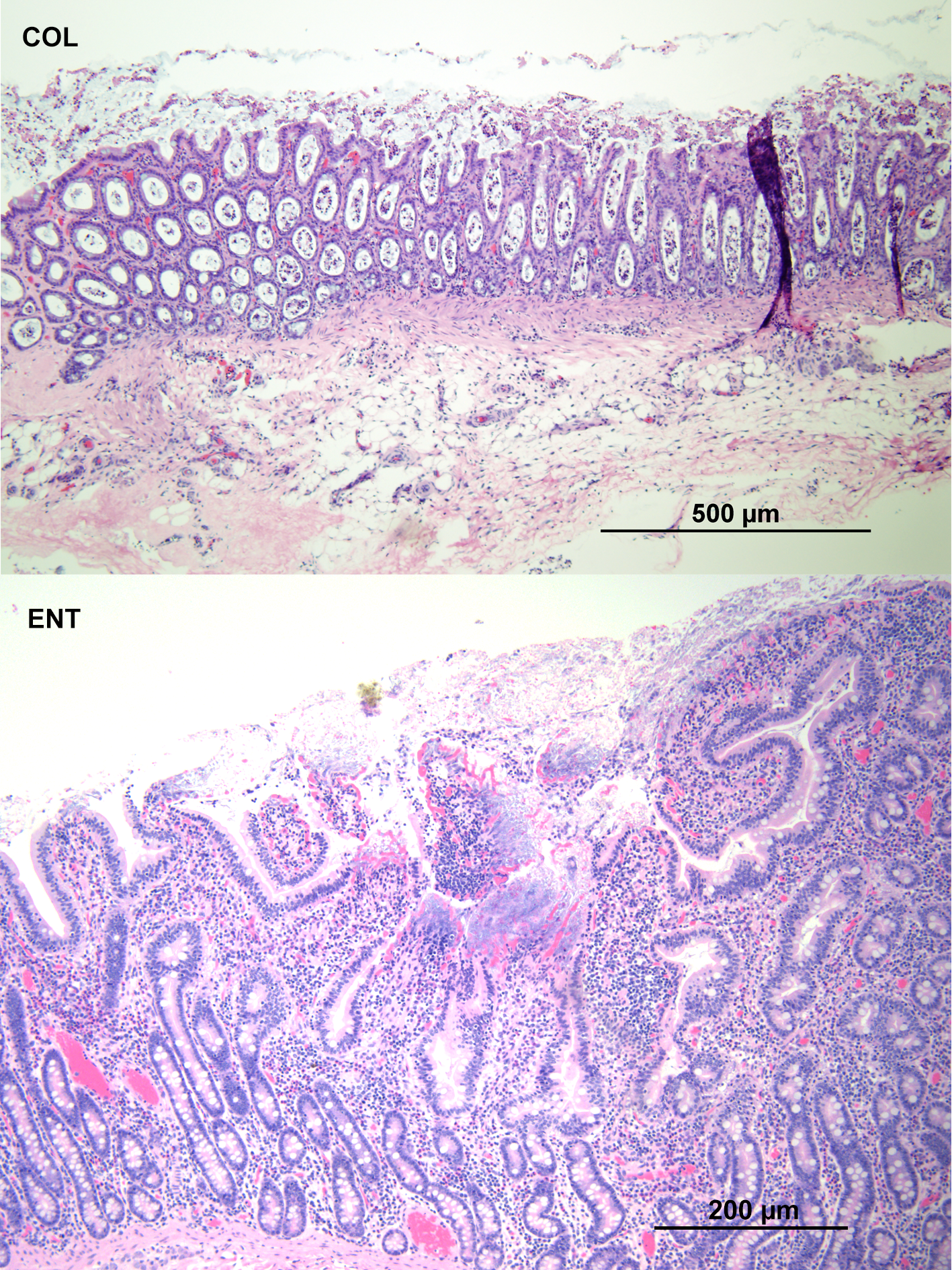

Supplement: Supplementary file 2 — Additional file 2: Figure 1. Representative H&E stained sections of formalin fixed colon (COL group) and ileum (ENT group) samples. [file 40813_2021_228_MOESM2_ESM.tif]
